# Auxin-mediated seed germination and crosstalk with other phytohormones

**DOI:** 10.3389/fpls.2025.1746472

**Published:** 2026-01-15

**Authors:** Anita Ament, Anna Kuchařová, Jovanka Vladejić, Jakub Bělíček, Federica Brunoni, Ondřej Novák

**Affiliations:** 1 Laboratory of Growth Regulators, Faculty of Science, Palacký University, Olomouc, Czechia; 2 Laboratory of Growth Regulators, Institute of Experimental Botany, The Czech Academy of Sciences, Olomouc, Czechia; 3 Department of Experimental Biology, Faculty of Science, Palacký University, Olomouc, Czechia

**Keywords:** abscisic acid, auxin, crosstalk, gibberellins, indole-3-acetic acid, phytohormones, seed germination

## Abstract

Seed germination is a critical and highly regulated process that transitions a dormant seed to an actively growing seedling. This process plays a vital physiological role in regulating seedling establishment, plant growth, and development, while ecologically it shapes species distribution patterns, drives plant population dynamics, and influences ecosystem productivity. Seed germination is tightly controlled by various environmental and intrinsic factors, with phytohormones acting as primary mediators. Auxins, mainly indole-3-acetic acid (IAA), are involved in many aspects of plant growth and development. Accumulating evidence suggests that IAA modulates the balance between dormancy and germination similarly to abscisic acid (ABA) and gibberellins (GAs). In this mini-review, we summarize our current knowledge on the molecular mechanisms underlying the modulatory roles of IAA during seed germination. We specifically examine the crosstalk between IAA and other key phytohormones (ABA and GAs) that shape germination outcomes. Clarifying these interactions will enhance our understanding of the dormancy-germination switch and may offer practical methods to control germination timing in agriculture.

## 1 Introduction

Seed germination is the developmental transition in which the metabolically quiescent embryo resumes growth and initiates seedling development (Bewley, 1997; Finch-Savage and Leubner-Metzger, 2006). Dormancy often serves as an adaptive mechanism, preventing germination under unfavorable conditions. Dormancy release is typically triggered by specific environmental cues, such as prolonged storage, optimal temperatures, or light, which reinitiate growth. Seeds perceive environmental signals and integrate them into endogenous signaling pathways through complex phytohormone networks to elicit downstream responses (e.g., dormancy and germination) (Kendall et al., 2011).

Abscisic acid (ABA) and gibberellins (GAs) are considered the main phytohormones regulating germination. In general, ABA suppresses seed germination, while GA promotes it. At the molecular level, this ABA-GA antagonism is modulated by complex transcriptional networks. ABA signaling is mediated by PYRABACTIN RESISTANCE (PYR)/REGULATORY COMPONENT OF ABSCISIC ACID RECEPTOR (RCAR) receptors that recognize the ABA molecule and inhibit 2C protein phosphatases (PP2Cs). This inhibition, in turn, activates SNF1-related kinases 2 (SnRK2s) and downstream transcription factors ABSCISIC ACID INSENSITIVE 3-5 (ABI3-5), which reinforce dormancy by repressing growth-promoting pathways (Park et al., 2009; Ma et al., 2009; Umezawa et al., 2009; Cutler et al., 2010). As dormancy is released, the expression of ABA biosynthetic genes declines, while catabolic genes, such as cytochrome P450 monooxygenase *CYP707A*, are upregulated, thereby reducing ABA levels (Okamoto et al., 2006). Concurrently, GA biosynthetic genes, including *GA 20-oxidases* (*GA20ox*) and *GA3ox*, are transcriptionally induced, leading to an increase in bioactive GA levels (Yamaguchi et al., 1998; Seo et al., 2006). Elevated GA levels trigger the proteasomal degradation of DELLA repressors, notably REPRESSOR OF GA1-3-LIKE2 (RGL2), lifting transcriptional constraints on germination-associated genes and enabling radicle protrusion (Dill et al., 2004; Tyler et al., 2004; Piskurewicz et al., 2008).

Auxin, primarily indole-3-acetic acid (IAA), is an important but complex regulator of seed germination (Wang et al., 2011; Liu et al., 2013; Wang et al., 2016; Ye et al., 2016; Shuai et al., 2017; Hussain et al., 2020; Mei et al., 2023; Tognacca et al., 2024). In *Arabidopsis thaliana*, IAA is produced mainly via the indole-3-pyruvic acid (IPyA) pathway in which TRYPTOPHAN AMINOTRANSFERASE OF ARABIDOPSIS (TAA) and YUCCA (YUC) flavin-containing monooxygenases convert tryptophan to IAA (Casanova-Sáez et al., 2021). Auxin distribution is shaped by polar transport: PIN-FORMED (PIN) efflux and AUXIN1/LIKE-AUX1 (AUX1/LAX) influx carriers establish auxin gradients that guide cell expansion and division (Luschnig and Friml, 2024). Auxin perception proceeds through both intracellular and extracellular routes (Vanneste et al., 2025). Canonically, auxin binding to TRANSPORT INHIBITOR RESPONSE1 (TIR1)/AUXIN-SIGNALING F-BOX (AFB) receptors promotes degradation of Aux/IAA repressors, releasing AUXIN RESPONSE FACTORS (ARFs) to activate transcription (Gray et al., 2001; Kepinski and Leyser, 2005; Tan et al., 2007). Several ARFs are further downregulated post-transcriptionally by microRNAs (miRNAs), refining auxin outputs (Liu et al., 2007).

Although many studies have probed how IAA interacts with the antagonistic balance between ABA and GA, the seed‐specific mechanisms by which auxin regulates germination have not been comprehensively determined. In this mini-review, we synthesize recent advances on auxin’s role in seed germination and its intricate crosstalk with other phytohormones, highlighting key mechanistic nodes and open questions that will direct future investigations.

## 2 Auxin gradients shape seed development

Auxin biosynthesis is rapidly induced upon fertilization of the central cell, driving early endosperm proliferation (Figueiredo et al., 2015; Batista et al., 2019; Guo et al., 2022). As development proceeds, auxin signaling intensifies during later stages of seed maturation (Pellizzaro et al., 2020). Auxin input and output reporters reveal that auxin response maxima emerge at the funiculus, chalaza, and micropylar integument. These maxima are established through localized TAA1-YUC biosynthesis in specific seed-coat domains and directed transport via PIN3 and AUX1 (Liu et al., 2023). These dynamics reflect coordinated changes in auxin supply, transport, and tissue sensitivity. Auxin signaling during development is associated with increased seed longevity (Pellizzaro et al., 2020) and, when elevated in the seed coat, larger seed size (Liu et al., 2023). Conversely, higher auxin activity in mature seeds is consistent with the maintenance of dormancy and delayed germination.

## 3 Application of exogenous IAA alters seed germination

Early evidence that auxin modulates germination came from exogenous applications of auxinic herbicides, which frequently delayed or inhibited seed germination, establishing that auxin-like activity can influence the germination process (Hamner et al., 1946; Hsueh and Lou, 1947). Subsequent work confirmed that high doses of exogenous IAA or its synthetic analogues delay germination in several species (Ramaih et al., 2003; Liu et al., 2013; Shuai et al., 2017). However, the underlying mechanisms are species-dependent. In Arabidopsis and soybean, inhibition is associated with the shifts in the ABA/GA balance. Exogenous IAA application upregulates ABA biosynthesis and signaling, limits ABA inactivation, while repressing GA biosynthesis and signaling (Liu et al., 2013; Shuai et al., 2017). For tobacco, soaking seeds in high IAA doses reduces the germination speed and can even induce secondary dormancy. In this case, the exogenous application of IAA does not alter ABA content, but instead, increases GA content, which the authors interpret as a compensatory response to counteract the auxin effect. Seeds then recover from dormancy as auxin levels subsequently decline (Li et al., 2016).

In contrast, low doses of IAA can promote seed germination in Arabidopsis (Wang et al., 2016). Seed priming with IAA has been shown to be beneficial in particular species, including cotton and Chinese fir, where it improves germination and seedling growth (Zhao and Zhong, 2013; Zhao et al., 2020), However, this effect was not observed in Arabidopsis (Ye et al., 2016). Auxin treatments can also counteract germination delays caused by salinity or drought in a dose-dependent manner (Ashraf and Foolad, 2005; Iqbal and Ashraf, 2007; Xing et al., 2023; Ellouzi et al., 2024). Consistent with these low-auxin effects, low amounts of IAA synthesized by plant growth-promoting rhizobacteria can also promote seed germination, enhance stress tolerance, and improve nutrient uptake (Fiodor et al., 2023; Shaffique et al., 2023).

Thus, auxin’s effect on seed germination can be either inhibitory or beneficial, depending on the dose, species, and physiological context.

## 4 Mutants in auxin metabolism, signaling, and transport show affected seed germination

Exogenous application of auxin alters germination in Arabidopsis, and lines overexpressing the *iaaM* gene, which encodes a bacterial enzyme that increases auxin production, also show severely delayed germination and deep primary dormancy. This observation suggests that perturbations in endogenous IAA can influence germination kinetics, and in turn raises the question of which steps in auxin metabolism are most crucial for seed germination. Disruption of the biosynthetic IPyA pathway, as seen in the *yuc1 yuc6* mutant, reduces auxin levels and leads to decreased dormancy and accelerated germination relative to the wild-type (Liu et al., 2013).

At the receptor level, loss of TIR1/AFB impairs Aux/IAA degradation and results in reduced auxin signaling. Loss-of-function *tir1*, *tir1 afb2*, *tir1 afb3*, and *tir1 afb1 afb2 afb3* mutants show an enhanced germination rate compared to wild-type, with the most substantial effect in the quadruple mutant under ABA supplementation (Liu et al., 2013). Auxin signaling provides another regulatory layer. Several *Aux/IAA* genes (*IAA1*, *IAA2*, *IAA3*, *IAA16*, *IAA20*, *IAA26*, *IAA28*, and *IAA29*) are highly expressed during germination (Winter et al., 2007; Carranco et al., 2010), suggesting functional relevance. Consistent with this, the loss-of-function *iaa8–1* mutant shows delayed radicle protrusion (Hussain et al., 2020). Conversely, gain-of-function mutants *axr2-1* (*IAA7*) and *axr3-1* (*IAA17*) carry mutations that reduce auxin-induced Aux/IAA protein degradation and germinate faster compared to wild-type. Mutants of downstream transcription factors, *arf10*, *arf16*, and the *arf10 arf16* double mutant, have enhanced germination and display ABA hyposensitivity compared to wild-type. In contrast, transgenic lines expressing miR160-resistant forms of *ARF10* and *ARF16* have the opposite effect (Liu et al., 2013).

Auxin transporters are also upregulated in non-dormant seeds compared to dormant ones, suggesting that, besides seed development, auxin redistribution plays a role in seed germination (Carrera et al., 2008). The loss-of-function *aux1–21* and *aux1–22* mutants exhibit slower germination compared to wild-type seeds (Wang et al., 2016). Under ABA treatment, germination is inhibited in both wild-type and *pin3-3*, *pin3-4*, *aux1-1*, *pin7-1*, *pin7–2* mutant seeds. However, mutants in auxin transport show significantly stronger inhibition compared to wild-type (Tognacca et al., 2024), underscoring the role of AUX1/PIN-mediated auxin fluxes in seed germination.

Together, these findings demonstrate that auxin influences germination through multiple regulatory layers, spanning biosynthesis, signaling, and transport (**Table 1**). Reduced auxin supply or impaired signaling often weakens dormancy, whereas elevated auxin levels reinforce it. Optimal spatial distribution of auxin, mediated by AUX1 and PIN transporters, further fine-tunes the timing of radicle emergence. This multilayered regulation sets the stage for understanding how auxin integrates with ABA and GA signals to control seed germination.

**Table 1:** Germination phenotype of Arabidopsis mutants in auxin metabolism and transport.

AGI gene code	Protein	Mutant allele	Disrupted process	Impact on seed germination	References
At4g32540, At5g25620	YUCCA proteins	*yuc1 yuc6*	Auxin biosynthesis	Enhanced germination	Liu et al., 2013
At3g62980	Auxin co-receptor F-box protein	*tir1*	Auxin perception	Enhanced germination under ABA supplementation	Liu et al., 2013
At4g03190	Auxin co-receptor F-box protein	*afb1*	Auxin perception	Enhanced germination under ABA supplementation	Liu et al., 2013
At3g26810	Auxin co-receptor F-box protein	*afb2*	Auxin perception	Enhanced germination under ABA supplementation	Liu et al., 2013
At1g12820	Auxin co-receptor F-box protein	*afb3*	Auxin perception	Enhanced germination under ABA supplementation	Liu et al., 2013
At3g62980, At3g26810	Auxin co-receptor F-box protein	*tir1 afb2*	Auxin perception	Enhanced germination, enhanced germination under ABA supplementation	Liu et al., 2013
At3g62980, At1g12820	Auxin co-receptor F-box protein	*tir1 afb3*	Auxin perception	Enhanced germination, enhanced germination under ABA supplementation	Liu et al., 2013
At4g03190, At3g26810, At1g12820, At3g62980	Auxin co-receptor F-box proteins	*tir1 afb1 afb2 afb3*	Auxin perception	Enhanced germination under ABA supplementation	Liu et al., 2013
At3g23050	Aux/IAA protein	*axr2-1*	Auxin signaling	Enhanced germination	Liu et al., 2013
At1g04240	Aux/IAA protein	*axr3-1*	Auxin signaling	Enhanced germination	Liu et al., 2013
At2g22670	Aux/IAA protein	*iaa8-1*	Auxin signaling	Delayed germination, delayed germination under NAA supplementation	Hussain et al., 2020
At2g28350	Auxin Response Factor	*arf10*	Auxin signaling	Enhanced germination under ABA supplementation	Liu et al., 2013
At4g30080	Auxin Response Factor	*arf16*	Auxin signaling	Enhanced germination under ABA supplementation	Liu et al., 2013
At2g28350, At4g30080	Auxin Response Factors	*arf10 arf16*	Auxin signaling	Enhanced germination, enhanced germination under ABA supplementation	Liu et al., 2013
At2g38120	Auxin influx carrier	*aux1-21*	Auxin influx	Delayed germination	Wang et al., 2016
At2g38120	Auxin influx carrier	*aux1-22*	Auxin influx	Delayed germination	Wang et al., 2016
At2g38120	Auxin influx carrier	*aux1-1*	Auxin influx	Delayed germination under ABA supplementation after red light pulse	Tognacca et al., 2024
At1g70940	Auxin efflux carrier	*pin3-3*	Auxin efflux	Delayed germination under ABA supplementation after red light pulse	Tognacca et al., 2024
At1g70940	Auxin efflux carrier	*pin3-4*	Auxin efflux	Delayed germination under ABA supplementation after red light pulse	Tognacca et al., 2024
At1g23080	Auxin efflux carrier	*pin7-1*	Auxin efflux	Delayed germination under ABA supplementation after red light pulse	Tognacca et al., 2024
At1g23080	Auxin efflux carrier	*pin7-2*	Auxin efflux	Delayed germination under ABA supplementation after red light pulse	Tognacca et al., 2024

## 5 Crosstalk between IAA and other phytohormones modulates seed germination

### 5.1 IAA and ABA

Crosstalk between auxin and ABA regulates hypocotyl elongation, root elongation, lateral root formation, and cotyledon growth (Emenecker and Strader, 2020). Beyond development, coordinated action of these two hormones also plays a critical role in abiotic stress responses, where their interaction modulates stress acclimation (Jing et al., 2023).

During seed germination, auxin closely interplays with ABA signaling to control radicle emergence. ABA is the central repressor of germination, and auxin reinforces this effect by modulating both ABA metabolism and downstream signaling. Physiological studies in soybean have shown that IAA treatment enhances the expression of ABA biosynthetic genes, such as *ABA2* and *AAO*, while simultaneously repressing ABA catabolism through *CYP707A1*, thereby elevating ABA levels (Shuai et al., 2017). At the signaling level, auxin promotes the expression of the transcription factors *ABI3*, *ABI4*, and *ABI5*, all of which are established repressors of germination (Liu et al., 2007, 2013; Shuai et al., 2017). Within this regulatory cascade, ABI3 acts upstream of ABI4 and ABI5, which in turn activate the transcription of *EARLY METHIONINE-LABELED 6* (*Em6*) and *Em1*. These genes encode hydrophilic proteins that stabilize cellular structures and confer desiccation tolerance, reinforcing seed dormancy (Söderman et al., 2000; Carles et al., 2002; Lopez-Molina et al., 2002; Skubacz et al., 2016).

Auxin influences the expression of *ABI* genes through the activity of Aux/IAA and ARF proteins during germination. Mutant of the Aux/IAA protein, IAA8 (*iaa8-1*), exhibits elevated transcript levels of *ABI3*, *ABI4*, and *ABI5* (Hussain et al., 2020). IAA8 associates with the *ABI3* promoter through unidentified ARFs, restricting ARF activity, which in turn reduces *ABI3* expression and promotes dormancy release (Hussain et al., 2020). By contrast, ARF family members show opposing effects on seed germination. ARF10/16 act as repressors of germination. Earlier work indicated that ARF10/16 act upstream of ABI3 (Liu et al., 2013). However, more recent studies demonstrated that they physically associate with ABI5, enhancing its transcriptional activity on downstream targets, and strengthening ABA-dependent repression of germination (Mei et al., 2023). Moreover, ARF16 was shown to interact with GERMOSTATIN RESISTANCE LOCUS 1 (GSR1) in a co-repressor complex during germination (Ye et al., 2016), pointing to broader regulatory networks. Conversely, ARF2 expression is induced by ABA. Overexpression of ARF2 alleviates ABA-mediated inhibition of germination by repressing the homeobox gene *HB33*, a known inhibitor of germination. Thus, ABA promotes ARF2 to dampen *HB33* expression, creating a negative feedback loop that limits ABA restraint and permits radicle emergence (Wang et al., 2011).

**Figure 1:**
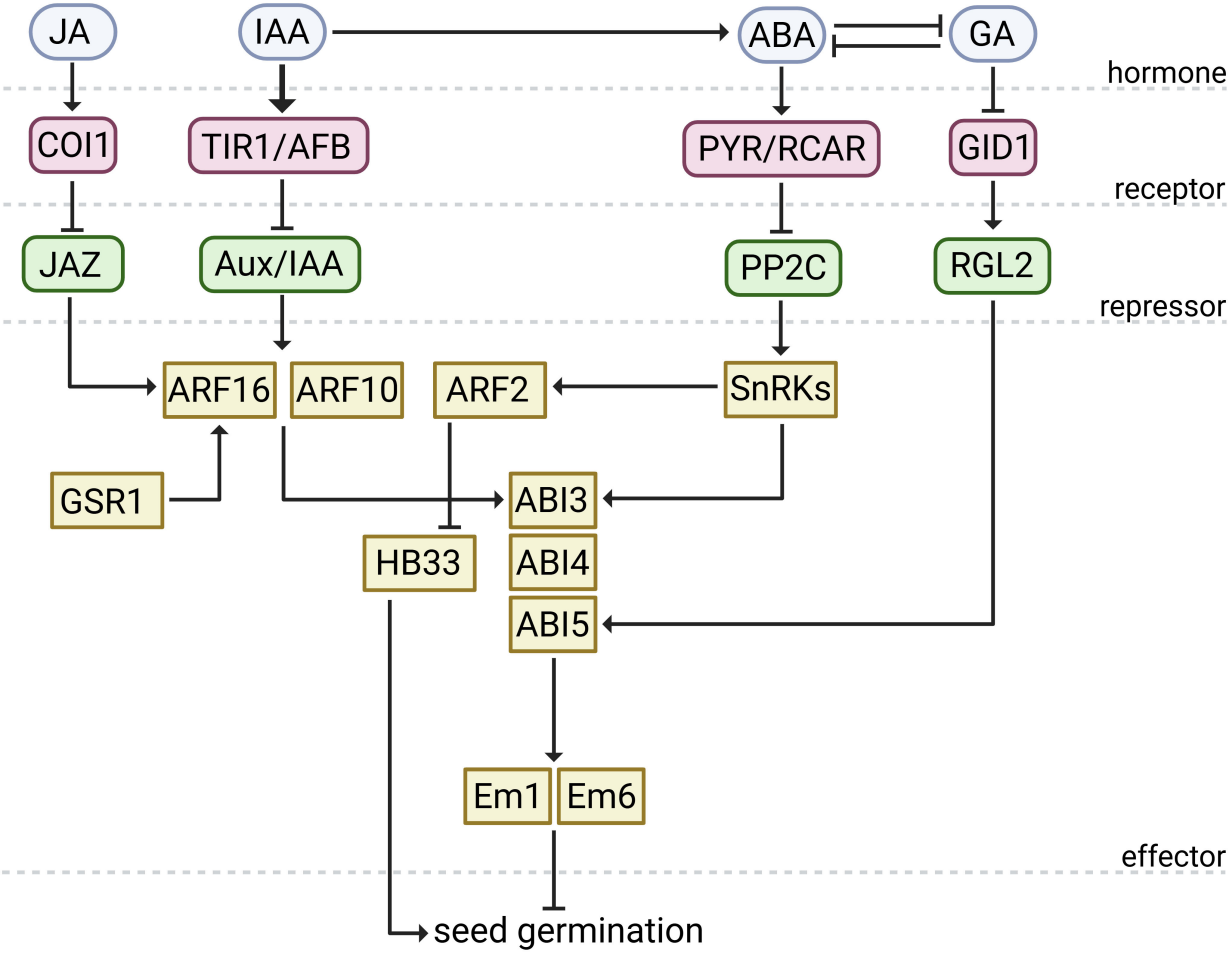
IAA-modulated seed germination is regulated by alterations in ABA and GA biosynthesis and/or signaling. IAA can promote ABA biosynthesis. ABA perception via PYR/RCAR blocks PP2Cs, releasing SnRK2 kinases that activate *ABI* and induce Em1/Em6, collectively restraining germination. Through TIR1/AFB, IAA triggers Aux/IAA degradation, releasing ARF10/ARF16 to enhance ABI3/ABI5 transcription of downstream targets and reinforce ABA-mediated inhibition. In parallel, ABA induces *ARF2*, which represses *HB33*. Because HB33 increases ABA sensitivity, ARF2-mediated repression of *HB33* can reduce ABA responsiveness, thereby permitting germination. IAA may antagonize GA accumulation, thereby limiting GID1-dependent removal of the DELLA repressor RGL2, which in turn strengthens ABI5 activity and blocks germination. Additional cues feed into the auxin module: JA-Ile perception via COI1 targets JAZ repressors for degradation, lifting their inhibition of the ARF10/16–ABI5 module. Freed ARF10/16 enhance ABI5’s transcriptional activity, reinforcing ABA signaling and thereby promoting seed dormancy. GSR1 forms a complex with ARF16 and acts as a co-repressor, inhibiting germination. Solid arrows indicate activation, and T-bars indicate suppression. Created in BioRender. Plíhal, O. (2026) https://BioRender.com/pfsp4tc.

Taken together, these findings reveal that auxin modulates seed germination at multiple levels, ultimately reinforcing ABA signaling to fine-tune the dormancy-to-germination transition (**Figure 1**). The contrasting roles of ARF2 versus ARF10/16 highlight auxin’s dual capacity to either attenuate or reinforce ABA signaling, providing flexibility in fine-tuning germination responses.

### 5.2 IAA, JA, and ABA

Wild-type seeds exposed to simultaneous treatment with ABA, IAA, and methyl jasmonate (MeJA) show dramatically lower germination rate than seeds treated with ABA/IAA or ABA/MeJA alone. This indicates that these three hormones act synergistically to reinforce ABA-mediated repression of germination (Mei et al., 2023). Consistently, mutants impaired in auxin biosynthesis, perception, or signaling display reduced responsiveness to ABA and MeJA treatments during germination (Liu et al., 2013; Mei et al., 2023), while JA signaling mutants display decreased responsiveness to ABA and IAA (Pan et al., 2020; Mei et al., 2023).

Several studies have revealed that ARF10/16 function as molecular bridges linking auxin and jasmonate (JA) signaling to ABA-dependent germination control. JA perception requires the CORONATINE INSENSITIVE 1 (COI1) receptor, which mediates the degradation of JAZ (JASMONATE ZIM-DOMAIN) repressors in response to the bioactive conjugate JA-isoleucine (JA-Ile) (Thines et al., 2007; Chini et al., 2007; Fonseca et al., 2009; Sheard et al., 2010). In this context, the *coi1–16* mutant shows reduced germination inhibition under ABA and MeJA treatment, a phenotype that can be partially rescued by ARF16 overexpression. This finding suggests that ARF16 could compensate for impaired JA perception, reinforcing the functional interplay between auxin, JA, and ABA during germination. At a molecular level, JAZ proteins act as negative regulators of the ARF10/16-ABI5 complex. When JA-Ile levels are low, JAZ proteins bind ARF10/16 and limit their capacity to enhance ABI5’s transcriptional function. JA-Ile perception triggers JAZ degradation, releasing ARF10/16 to potentiate ABI5 activity (Mei et al., 2023). This ARF-JAZ-ABI5 regulatory module thus emerges as a central hub integrating IAA and JA signals into ABA-dependent repression of seed germination (**Figure 1**).

### 5.3 IAA and GA

GA, together with ABA, mediates the transition from dormancy to germination. Upon imbibition, GA-biosynthetic genes *GA20ox* and *GA3ox* are transcriptionally induced, increasing bioactive GA levels (Yamaguchi et al., 1998; Seo et al., 2006). GA binding to the receptor GA INSENSITIVE DWARF1 (GID1) promotes the formation of GID1-DELLA complex and its ubiquitination, triggering 26S proteasome degradation of DELLA repressors. In seeds, RGL2 is a key DELLA repressor acting upstream of ABI5 (Piskurewicz et al., 2008). The removal of DELLAs lifts transcriptional repression on germination-associated genes and enables radicle protrusion.

Crosstalk between IAA and GA regulates a broad suite of developmental processes, including hypocotyl elongation, root meristem maintenance and elongation, fruit initiation, and early fruit development (He and Yamamuro, 2022; Shtin et al., 2022; Krahmer and Fankhauser, 2024). How this crosstalk operates during germination is less clear. Across vegetative and fruit tissues, IAA promotes GA synthesis by activating GA biosynthetic genes and modulating GA catabolism genes in Arabidopsis, rice, and pea (Frigerio et al., 2006; Yin et al., 2007; O’Neill et al., 2010). During soybean germination, however, high exogenous IAA shifts the balance toward ABA, downregulating the transcription of *GA3ox1* and *GA3ox2*, and repressing GA-signaling transduction by upregulating *DELLA* gene expression (Shuai et al., 2017). Mechanistically, IAA-GA crosstalk in non-seed tissues operates via canonical Aux/IAA-ARF modules. In tomato fruit, ARF7-IAA9 and DELLA/PROCERA co-regulate the transcription of GA-biosynthetic genes (Hu et al., 2018), while in Arabidopsis roots, auxin is required for GA-triggered degradation of the DELLA proteins (Fu and Harberd, 2003). Whether a similar process operates during germination remains to be elucidated (**Figure 1**). GA can also influence auxin transport capacity. In seedlings, GA helps to maintain PIN abundance and trafficking, and GA deficiency reduces PIN-dependent transport (Willige et al., 2011). In seeds, *AUX1*, *PIN3*, and *PIN7* are responsive to GA cues, and loss-of-function mutants show altered germination kinetics under ABA supplementation (Tognacca et al., 2024). However, a direct GA-*AUX1*/*PIN3*/*PIN7* regulatory link during germination has not yet been demonstrated.

## 6 Spatial IAA distribution regulates seed germination

Germination succeeds when the growing embryo generates enough force to overcome the mechanical resistance of the testa and micropylar endosperm. GA promotes the embryo’s growth potential and endosperm loosening, whereas ABA reinforces restraint. Consequently, the spatial distribution of hormones across the embryo and endosperm is critical to timing radicle emergence (Chandrasekaran et al., 2022; Zhao et al., 2022; Matilla, 2025). There is increasing evidence that auxin distribution within the embryo influences germination. At the transcriptional level, GA upregulates, while ABA downregulates the expression of auxin transporters *PIN3*, *PIN7*, and *AUX1* (Ogawa et al., 2003; Nakabayashi et al., 2005; Penfield et al., 2006). Functionally, chemical inhibition of polar auxin transport or loss-of-function mutations in *aux1*, *pin3*, or *pin7* slow germination and increase ABA sensitivity (Tognacca et al., 2024). During imbibition, ABA levels decline, whereas IAA often rises under germination-promoting conditions, such as red light (Preston et al., 2009; Tognacca et al., 2024). Accordingly, directed auxin transport must redistribute IAA within the seed compartments. AUX1 facilitates auxin delivery to the radicle tip, and seed-specific AUX1 overexpression increases radicle tip cell number and accelerates germination compared with wild-type (Wang et al., 2016). Consistent with this, Tognacca et al. (2024) proposed that red-light induction of *AUX1*, *PIN3*, and *PIN7* enhances auxin delivery toward the radicle tip, helping establish a permissive auxin gradient across the embryonic-axis elongation zone and thereby facilitating radicle emergence. Thus, the precise routing of IAA within embryonic tissues is a modulatory step in seed germination, cooperating with GA to enhance embryo growth potential while counteracting the restraint imposed by ABA.

## 7 Conclusion

Auxin’s effect on seed germination is both dose- and context-dependent. In Arabidopsis, high auxin concentrations generally delay germination, while low exogenous doses can promote it. Elevated IAA supply via the IPyA pathway increases ARF-dependent transcription and reinforces ABA-dependent dormancy. Whether alternative auxin biosynthetic routes exert similar effects in seeds remains unknown and warrants further investigation.

At the signaling level, ARF functions are dual: ARF10/16 strengthen dormancy by acting through ABI transcription factors and, via crosstalk with JA, assemble an ARF10/16-JAZ-ABI5 regulatory node. In contrast, ARF2 tends to alleviate ABA-mediated inhibition, enabling radicle protrusion. While auxin clearly modifies GA levels in germinating seeds, whether it directly targets GA signaling components remains unclear. In non-seed contexts, Aux/IAA-ARF modules can condition DELLA turnover. Whether analogous seed-specific ARF-DELLA links operate requires demonstration. A plausible, and not mutually exclusive, alternative is that apparent IAA-GA interactions in seeds act indirectly through auxin’s reinforcement of ABA signaling and the established ABA-GA feedback circuitry.

Beyond total hormone levels, AUX1/PIN-mediated auxin routing helps set the timing of radicle protrusion. Local auxin distribution and its interplay with ABA and GA across the embryonic axis and micropylar endosperm likely predict emergence more accurately than bulk hormone levels. This highlights the need for building spatiotemporal maps of IAA, GA, and ABA at cellular resolution to determine when, where, and how much of each phytohormone signal is required to overcome endosperm resistance. Given that auxin can affect germination via biosynthesis, signaling, and transport pathways, it is also reasonable to ask whether auxin inactivation and catabolism tune the kinetics of radicle emergence.

Clarifying how auxin integrates with ABA and GA during germination will enhance our understanding of the dormancy-germination switch and facilitate the development of practical methods to control germination timing and uniformity in agriculturally important crops.

## References

[B1] Ashraf M. Foolad M. R. (2005). Pre-sowing seed treatment—A shotgun approach to improve germination, plant growth, and crop yield under saline and non-saline conditions. Adv. Agron. 88, 223–271. doi: 10.1016/S0065-2113(05)88006-X

[B2] Batista R. A. Figueiredo D. D. Santos-González J. Köhler C. (2019). Auxin regulates endosperm cellularization in Arabidopsis. Genes Dev. 33, 466–476. doi: 10.1101/gad.316554.118, PMID: 30819818 PMC6446538

[B3] Bewley J. D. (1997). Seed germination and dormancy. Plant Cell 9, 1055–1066. doi: 10.1105/tpc.9.7.1055, PMID: 12237375 PMC156979

[B4] Carles C. Bies-Etheve N. Aspart L. Léon-Kloosterziel K. M. Koornneef M. Echeverria M. . (2002). Regulation of Arabidopsis thaliana Em genes: role of ABI5. Plant J. 30, 373–383. doi: 10.1046/j.1365-313X.2002.01295.x, PMID: 12000684

[B5] Carranco R. Espinosa J. M. Prieto-Dapena P. Almoguera C. Jordano J. (2010). Repression by an auxin/indole acetic acid protein connects auxin signaling with heat shock factor-mediated seed longevity. Proc. Natl. Acad. Sci. U.S.A. 107, 21908–21913. doi: 10.1073/pnas.1014856107, PMID: 21115822 PMC3003009

[B6] Carrera E. Holman T. Medhurst A. Dietrich D. Footitt S. Theodoulou F. L. . (2008). Seed after-ripening is a discrete developmental pathway associated with specific gene networks in Arabidopsis. Plant J. 53, 214–224. doi: 10.1111/j.1365-313X.2007.03331.x, PMID: 18028281 PMC2254144

[B7] Casanova-Sáez R. Mateo-Bonmatí E. Ljung K. (2021). Auxin metabolism in plants. Cold Spring Harb. Perspect. Biol. 13, a039867. doi: 10.1101/cshperspect.a039867, PMID: 33431579 PMC7919392

[B8] Chandrasekaran U. Zhao X. Luo X. Wei S. Shu K. (2022). Endosperm weakening: The gateway to a seed’s new life. Plant Physiol. Biochem. 178, 31–39. doi: 10.1016/j.plaphy.2022.02.016, PMID: 35276594

[B9] Chini A. Fonseca S. Fernández G. Adie B. Chico J. M. Lorenzo O. . (2007). The JAZ family of repressors is the missing link in jasmonate signalling. Nature 448, 666–671. doi: 10.1038/nature06006, PMID: 17637675

[B10] Cutler S. R. Rodriguez P. L. Finkelstein R. R. Abrams S. R. (2010). Abscisic acid: emergence of a core signaling network. Annu. Rev. Plant Biol. 61, 651–679. doi: 10.1146/annurev-arplant-042809-112122, PMID: 20192755

[B11] Dill A. Thomas S. G. Hu J. Steber C. M. Sun T. (2004). The arabidopsis F-box protein SLEEPY1 targets gibberellin signaling repressors for gibberellin-induced degradation[W. Plant Cell 16, 1392–1405. doi: 10.1105/tpc.020958, PMID: 15155881 PMC490034

[B12] Ellouzi H. Ben Slimene Debez I. Amraoui S. Rabhi M. Hanana M. Alyami N. M. . (2024). Effect of seed priming with auxin on ROS detoxification and carbohydrate metabolism and their relationship with germination and early seedling establishment in salt stressed maize. BMC Plant Biol. 24, 704. doi: 10.1186/s12870-024-05413-w, PMID: 39054427 PMC11270924

[B13] Emenecker R. J. Strader L. C. (2020). Auxin-abscisic acid interactions in plant growth and development. Biomolecules 10, 281. doi: 10.3390/biom10020281, PMID: 32059519 PMC7072425

[B14] Figueiredo D. D. Batista R. A. Roszak P. J. Köhler C. (2015). Auxin production couples endosperm development to fertilization. Nat. Plants 1, 15184. doi: 10.1038/nplants.2015.184, PMID: 27251719

[B15] Finch-Savage W. E. Leubner-Metzger G. (2006). Seed dormancy and the control of germination. New Phytol. 171, 501–523. doi: 10.1111/j.1469-8137.2006.01787.x, PMID: 16866955

[B16] Fiodor A. Ajijah N. Dziewit L. Pranaw K. (2023). Biopriming of seed with plant growth-promoting bacteria for improved germination and seedling growth. Front. Microbiol. 14. doi: 10.3389/fmicb.2023.1142966, PMID: 36925481 PMC10011460

[B17] Fonseca S. Chini A. Hamberg M. Adie B. Porzel A. Kramell R. . (2009). (+)-7-iso-Jasmonoyl-L-isoleucine is the endogenous bioactive jasmonate. Nat. Chem. Biol. 5, 344–350. doi: 10.1038/nchembio.161, PMID: 19349968

[B18] Frigerio M. Alabadí D. Pérez-Gómez J. García-Cárcel L. Phillips A. L. Hedden P. . (2006). Transcriptional regulation of gibberellin metabolism genes by auxin signaling in arabidopsis. Plant Physiol. 142, 553–563. doi: 10.1104/pp.106.084871, PMID: 16905669 PMC1586059

[B19] Fu X. Harberd N. P. (2003). Auxin promotes Arabidopsis root growth by modulating gibberellin response. Nature 421, 740–743. doi: 10.1038/nature01387, PMID: 12610625

[B20] Gray W. M. Kepinski S. Rouse D. Leyser O. Estelle M. (2001). Auxin regulates SCFTIR1-dependent degradation of AUX/IAA proteins. Nature 414, 271–276. doi: 10.1038/35104500, PMID: 11713520

[B21] Guo L. Luo X. Li M. Joldersma D. Plunkert M. Liu Z. (2022). Mechanism of fertilization-induced auxin synthesis in the endosperm for seed and fruit development. Nat. Commun. 13, 3985. doi: 10.1038/s41467-022-31656-y, PMID: 35810202 PMC9271072

[B22] Hamner C. L. Moulton J. E. Tukey H. B. (1946). Effect of treating soil and seeds with 2,4-dichlorophenoxyacetic acid on germination and development of seedlings. Botanical Gazette 107, 352–361. doi: 10.1086/335358

[B23] He H. Yamamuro C. (2022). Interplays between auxin and GA signaling coordinate early fruit development. Horticulture Res. 9, uhab078. doi: 10.1093/hr/uhab078, PMID: 35043212 PMC8955447

[B24] Hsueh Y. L. Lou C. H. (1947). Effects of 2,4-D on seed germination and respiration. Science 105, 283–285. doi: 10.1126/science.105.2724.283, PMID: 17835144

[B25] Hu J. Israeli A. Ori N. Sun T. (2018). The interaction between DELLA and ARF/IAA mediates crosstalk between gibberellin and auxin signaling to control fruit initiation in tomato. Plant Cell 30, 1710–1728. doi: 10.1105/tpc.18.00363, PMID: 30008445 PMC6139683

[B26] Hussain S. Kim S. H. Bahk S. Ali A. Nguyen X. C. Yun D.-J. . (2020). The auxin signaling repressor IAA8 promotes seed germination through down-regulation of ABI3 transcription in arabidopsis. Front. Plant Sci. 11. doi: 10.3389/fpls.2020.00111, PMID: 32153614 PMC7045070

[B27] Iqbal M. Ashraf M. (2007). Seed treatment with auxins modulates growth and ion partitioning in salt-stressed wheat plants. JIPB 49, 1003–1015. doi: 10.1111/j.1672-9072.2007.00488.x

[B28] Jing H. Wilkinson E. G. Sageman-Furnas K. Strader L. C. (2023). Auxin and abiotic stress responses. J. Exp. Bot. 74, 7000–7014. doi: 10.1093/jxb/erad325, PMID: 37591508 PMC10690732

[B29] Kendall S. L. Hellwege A. Marriot P. Whalley C. Graham I. A. Penfield S. (2011). Induction of dormancy in *arabidopsis* summer annuals requires parallel regulation of *DOG1* and hormone metabolism by low temperature and CBF transcription factors. Plant Cell 23, 2568–2580. doi: 10.1105/tpc.111.087643, PMID: 21803937 PMC3226211

[B30] Kepinski S. Leyser O. (2005). The Arabidopsis F-box protein TIR1 is an auxin receptor. Nature 435, 446–451. doi: 10.1038/nature03542, PMID: 15917798

[B31] Krahmer J. Fankhauser C. (2024). Environmental control of hypocotyl elongation. Annu. Rev. Plant Biol. 75, 489–519. doi: 10.1146/annurev-arplant-062923-023852, PMID: 38012051

[B32] Li Z. Zhang J. Liu Y. Zhao J. Fu J. Ren X. . (2016). Exogenous auxin regulates multi-metabolic network and embryo development, controlling seed secondary dormancy and germination in Nicotiana tabacum L. BMC Plant Biol. 16, 41. doi: 10.1186/s12870-016-0724-5, PMID: 26860357 PMC4748683

[B33] Liu H. Luo Q. Tan C. Song J. Zhang T. Men S. (2023). Biosynthesis- and transport-mediated dynamic auxin distribution during seed development controls seed size in Arabidopsis. Plant J. 113, 1259–1277. doi: 10.1111/tpj.16109, PMID: 36648165

[B34] Liu P. Montgomery T. A. Fahlgren N. Kasschau K. D. Nonogaki H. Carrington J. C. (2007). Repression of AUXIN RESPONSE FACTOR10 by microRNA160 is critical for seed germination and post-germination stages. Plant J. 52, 133–146. doi: 10.1111/j.1365-313X.2007.03218.x, PMID: 17672844

[B35] Liu X. Zhang H. Zhao Y. Feng Z. Li Q. Yang H.-Q. . (2013). Auxin controls seed dormancy through stimulation of abscisic acid signaling by inducing ARF-mediated ABI3 activation in Arabidopsis. Proc. Natl. Acad. Sci. U.S.A. 110, 15485–15490. doi: 10.1073/pnas.1304651110, PMID: 23986496 PMC3780901

[B36] Lopez-Molina L. Mongrand S. McLachlin D. T. Chait B. T. Chua N. (2002). ABI5 acts downstream of ABI3 to execute an ABA-dependent growth arrest during germination. Plant J. 32, 317–328. doi: 10.1046/j.1365-313X.2002.01430.x, PMID: 12410810

[B37] Luschnig C. Friml J. (2024). Over 25 years of decrypting PIN-mediated plant development. Nat. Commun. 15, 9904. doi: 10.1038/s41467-024-54240-y, PMID: 39548100 PMC11567971

[B38] Ma Y. Szostkiewicz I. Korte A. Moes D. Yang Y. Christmann A. . (2009). Regulators of PP2C phosphatase activity function as abscisic acid sensors. Science 324, 1064–1068. doi: 10.1126/science.1172408, PMID: 19407143

[B39] Matilla A. J. (2025). Seed germination compromises expansion pressure, cell wall alterations, and the cuticular layer: New insights. Plant Sci. 359, 112612. doi: 10.1016/j.plantsci.2025.112612, PMID: 40518019

[B40] Mei S. Zhang M. Ye J. Du J. Jiang Y. Hu Y. (2023). Auxin contributes to jasmonate-mediated regulation of abscisic acid signaling during seed germination in Arabidopsis. Plant Cell 35, 1110–1133. doi: 10.1093/plcell/koac362, PMID: 36516412 PMC10015168

[B41] Nakabayashi K. Okamoto M. Koshiba T. Kamiya Y. Nambara E. (2005). Genome-wide profiling of stored mRNA in Arabidopsis thaliana seed germination: epigenetic and genetic regulation of transcription in seed. Plant J. 41, 697–709. doi: 10.1111/j.1365-313X.2005.02337.x, PMID: 15703057

[B42] O’Neill D. P. Davidson S. E. Clarke V. C. Yamauchi Y. Yamaguchi S. Kamiya Y. . (2010). Regulation of the gibberellin pathway by auxin and DELLA proteins. Planta 232, 1141–1149. doi: 10.1007/s00425-010-1248-0, PMID: 20706734

[B43] Ogawa M. Hanada A. Yamauchi Y. Kuwahara A. Kamiya Y. Yamaguchi S. (2003). Gibberellin biosynthesis and response during arabidopsis seed germination. Plant Cell 15, 1591–1604. doi: 10.1105/tpc.011650, PMID: 12837949 PMC165403

[B44] Okamoto M. Kuwahara A. Seo M. Kushiro T. Asami T. Hirai N. . (2006). CYP707A1 and CYP707A2, which encode abscisic acid 8′-hydroxylases, are indispensable for proper control of seed dormancy and germination in arabidopsis. Plant Physiol. 141, 97–107. doi: 10.1104/pp.106.079475, PMID: 16543410 PMC1459320

[B45] Pan J. Hu Y. Wang H. Guo Q. Chen Y. Howe G. A. . (2020). Molecular mechanism underlying the synergetic effect of jasmonate on abscisic acid signaling during seed germination in arabidopsis. Plant Cell 32, 3846–3865. doi: 10.1105/tpc.19.00838, PMID: 33023956 PMC7721325

[B46] Park S.-Y. Fung P. Nishimura N. Jensen D. R. Fujii H. Zhao Y. . (2009). Abscisic acid inhibits type 2C protein phosphatases via the PYR/PYL family of START proteins. Science 324, 1068–1071. doi: 10.1126/science.1173041, PMID: 19407142 PMC2827199

[B47] Pellizzaro A. Neveu M. Lalanne D. Ly Vu B. Kanno Y. Seo M. . (2020). A role for auxin signaling in the acquisition of longevity during seed maturation. New Phytol. 225, 284–296. doi: 10.1111/nph.16150, PMID: 31461534

[B48] Penfield S. Li Y. Gilday A. D. Graham S. Graham I. A. (2006). Arabidopsis ABA INSENSITIVE4 regulates lipid mobilization in the embryo and reveals repression of seed germination by the endosperm. Plant Cell 18, 1887–1899. doi: 10.1105/tpc.106.041277, PMID: 16844907 PMC1533976

[B49] Piskurewicz U. Jikumaru Y. Kinoshita N. Nambara E. Kamiya Y. Lopez-Molina L. (2008). The gibberellic acid signaling repressor RGL2 inhibits arabidopsis seed germination by stimulating abscisic acid synthesis and ABI5 activity. Plant Cell 20, 2729–2745. doi: 10.1105/tpc.108.061515, PMID: 18941053 PMC2590721

[B50] Preston J. Tatematsu K. Kanno Y. Hobo T. Kimura M. Jikumaru Y. . (2009). Temporal Expression Patterns of Hormone Metabolism Genes during Imbibition of Arabidopsis thaliana Seeds: A Comparative Study on Dormant and Non-Dormant Accessions. Plant Cell Physiol. 50, 1786–1800. doi: 10.1093/pcp/pcp121, PMID: 19713425

[B51] Ramaih S. Guedira M. Paulsen G. M. (2003). Relationship of indoleacetic acid and tryptophan to dormancy and preharvest sprouting of wheat. Funct. Plant Biol. 30, 939. doi: 10.1071/FP03113, PMID: 32689078

[B52] Seo M. Hanada A. Kuwahara A. Endo A. Okamoto M. Yamauchi Y. . (2006). Regulation of hormone metabolism in Arabidopsis seeds: phytochrome regulation of abscisic acid metabolism and abscisic acid regulation of gibberellin metabolism. Plant J. 48, 354–366. doi: 10.1111/j.1365-313X.2006.02881.x, PMID: 17010113

[B53] Shaffique S. Imran M. Kang S.-M. Khan M. A. Asaf S. Kim W.-C. . (2023). Seed Bio-priming of wheat with a novel bacterial strain to modulate drought stress in Daegu, South Korea. Front. Plant Sci. 14. doi: 10.3389/fpls.2023.1118941, PMID: 37180396 PMC10173886

[B54] Sheard L. B. Tan X. Mao H. Withers J. Ben-Nissan G. Hinds T. R. . (2010). Jasmonate perception by inositol-phosphate-potentiated COI1–JAZ co-receptor. Nature 468, 400–405. doi: 10.1038/nature09430, PMID: 20927106 PMC2988090

[B55] Shtin M. Dello Ioio R. Del Bianco M. (2022). It’s time for a change: the role of gibberellin in root meristem development. Front. Plant Sci. 13. doi: 10.3389/fpls.2022.882517, PMID: 35592570 PMC9112047

[B56] Shuai H. Meng Y. Luo X. Chen F. Zhou W. Dai Y. . (2017). Exogenous auxin represses soybean seed germination through decreasing the gibberellin/abscisic acid (GA/ABA) ratio. Sci. Rep. 7, 12620. doi: 10.1038/s41598-017-13093-w, PMID: 28974733 PMC5626727

[B57] Skubacz A. Daszkowska-Golec A. Szarejko I. (2016). The role and regulation of ABI5 (ABA-insensitive 5) in plant development, abiotic stress responses and phytohormone crosstalk. Front. Plant Sci. 7. doi: 10.3389/fpls.2016.01884, PMID: 28018412 PMC5159420

[B58] Söderman E. M. Brocard I. M. Lynch T. J. Finkelstein R. R. (2000). Regulation and function of the arabidopsis ABA-insensitive4 gene in seed and abscisic acid response signaling networks. Plant Physiol. 124, 1752–1765. doi: 10.1104/pp.124.4.1752, PMID: 11115891 PMC59872

[B59] Tan X. Calderon-Villalobos L. I. A. Sharon M. Zheng C. Robinson C. V. Estelle M. . (2007). Mechanism of auxin perception by the TIR1 ubiquitin ligase. Nature 446, 640–645. doi: 10.1038/nature05731, PMID: 17410169

[B60] Thines B. Katsir L. Melotto M. Niu Y. Mandaokar A. Liu G. . (2007). JAZ repressor proteins are targets of the SCFCOI1 complex during jasmonate signalling. Nature 448, 661–665. doi: 10.1038/nature05960, PMID: 17637677

[B61] Tognacca R. S. Ljung K. Botto J. F. (2024). Unveiling molecular signatures in light-induced seed germination: insights from PIN3, PIN7, and AUX1 in arabidopsis thaliana. Plants 13, 408. doi: 10.3390/plants13030408, PMID: 38337941 PMC10856848

[B62] Tyler L. Thomas S. G. Hu J. Dill A. Alonso J. M. Ecker J. R. . (2004). DELLA proteins and gibberellin-regulated seed germination and floral development in arabidopsis. Plant Physiol. 135, 1008–1019. doi: 10.1104/pp.104.039578, PMID: 15173565 PMC514135

[B63] Umezawa T. Sugiyama N. Mizoguchi M. Hayashi S. Myouga F. Yamaguchi-Shinozaki K. . (2009). Type 2C protein phosphatases directly regulate abscisic acid-activated protein kinases in Arabidopsis. Proc. Natl. Acad. Sci. U.S.A. 106, 17588–17593. doi: 10.1073/pnas.0907095106, PMID: 19805022 PMC2754379

[B64] Vanneste S. Pei Y. Friml J. (2025). Mechanisms of auxin action in plant growth and development. Nat. Rev. Mol. Cell Biol. 26, 648–666. doi: 10.1038/s41580-025-00851-2, PMID: 40389696

[B65] Wang Z. Chen F. Li X. Cao H. Ding M. Zhang C. . (2016). Arabidopsis seed germination speed is controlled by SNL histone deacetylase-binding factor-mediated regulation of AUX1. Nat. Commun. 7, 13412. doi: 10.1038/ncomms13412, PMID: 27834370 PMC5114640

[B66] Wang L. Hua D. He J. Duan Y. Chen Z. Hong X. . (2011). Auxin response factor2 (ARF2) and its regulated homeodomain gene HB33 mediate abscisic acid response in arabidopsis. PloS Genet. 7, e1002172. doi: 10.1371/journal.pgen.1002172, PMID: 21779177 PMC3136439

[B67] Willige B. C. Isono E. Richter R. Zourelidou M. Schwechheimer C. (2011). Gibberellin regulates PIN-FORMED abundance and is required for auxin transport–dependent growth and development in arabidopsis thaliana. Plant Cell 23, 2184–2195. doi: 10.1105/tpc.111.086355, PMID: 21642547 PMC3160035

[B68] Winter D. Vinegar B. Nahal H. Ammar R. Wilson G. V. Provart N. J. (2007). An “Electronic fluorescent pictograph” Browser for exploring and analyzing large-scale biological data sets. PloS One 2, e718. doi: 10.1371/journal.pone.0000718, PMID: 17684564 PMC1934936

[B69] Xing X. Cao C. Li S. Wang H. Xu Z. Qi Y. . (2023). α-naphthaleneacetic acid positively regulates soybean seed germination and seedling establishment by increasing antioxidant capacity, triacylglycerol mobilization and sucrose transport under drought stress. Plant Physiol. Biochem. 201, 107890. doi: 10.1016/j.plaphy.2023.107890, PMID: 37454467

[B70] Yamaguchi S. Smith M. W. Brown R. G. S. Kamiya Y. Sun T. (1998). Phytochrome regulation and differential expression of gibberellin 3b-hydroxylase genes in germinating arabidopsis seeds. Plant Cell 10, 2115. doi: 10.2307/3870788, PMID: 9836749 PMC143973

[B71] Ye Y. Gong Z. Lu X. Miao D. Shi J. Lu J. . (2016). Germostatin resistance locus 1 encodes a PHD finger protein involved in auxin-mediated seed dormancy and germination. Plant J. 85, 3–15. doi: 10.1111/tpj.13086, PMID: 26611158

[B72] Yin C. Gan L. Ng D. Zhou X. Xia K. (2007). Decreased panicle-derived indole-3-acetic acid reduces gibberellin A1 level in the uppermost internode, causing panicle enclosure in male sterile rice Zhenshan 97A. J. Exp. Bot. 58, 2441–2449. doi: 10.1093/jxb/erm077, PMID: 17556768

[B73] Zhao T. Deng X. Xiao Q. Han Y. Zhu S. Chen J. (2020). IAA priming improves the germination and seedling growth in cotton (Gossypium hirsutum L.) via regulating the endogenous phytohormones and enhancing the sucrose metabolism. Ind. Crops Products 155, 112788. doi: 10.1016/j.indcrop.2020.112788

[B74] Zhao H. Zhang Y. Zheng Y. (2022). Integration of ABA, GA, and light signaling in seed germination through the regulation of ABI5. Front. Plant Sci. 13. doi: 10.3389/fpls.2022.1000803, PMID: 36092418 PMC9449724

[B75] Zhao G. Zhong T. (2013). Influence of exogenous IAA and GA on seed germination, vigor and their endogenous levels in Cunninghamia lanceolata. Scandinavian J. For. Res. 28, 511–517. doi: 10.1080/02827581.2013.783099

